# LLL-3 inhibits STAT3 activity, suppresses glioblastoma cell growth and prolongs survival in a mouse glioblastoma model

**DOI:** 10.1038/sj.bjc.6604793

**Published:** 2009-01-06

**Authors:** B Fuh, M Sobo, L Cen, D Josiah, B Hutzen, K Cisek, D Bhasin, N Regan, L Lin, C Chan, H Caldas, S DeAngelis, C Li, P-K Li, J Lin

**Affiliations:** 1 Department of Pediatrics, BSOM, East Carolina University, Greenville, NC 27834, USA; 2 Department of Pediatrics, Center for Childhood Cancer, The Research Institute at Nationwide Children's Hospital, College of Medicine, The Ohio State University, Columbus, OH 43205, USA; 3 Department of Neurosurgery, Brain Tumor Center of Excellence, Wake Forest University Health Sciences, Winston-Salem, NC 27157, USA; 4 Biophysics Graduate Program, the Ohio State University, Columbus, OH 43210, USA; 5 Division of Medicinal Chemistry and Pharmacology, College of Pharmacy, The Ohio State University, Columbus, OH 43210, USA

**Keywords:** STAT3, apoptosis, glioblastoma, LLL-3

## Abstract

Persistent activation of the signal transducer and activator of transcription 3 (STAT3) signalling has been linked to oncogenesis and the development of chemotherapy resistance in glioblastoma and other cancers. Inhibition of the STAT3 pathway thus represents an attractive therapeutic approach for cancer. In this study, we investigated the inhibitory effects of a small molecule compound known as LLL-3, which is a structural analogue of the earlier reported STAT3 inhibitor, STA-21, on the cell viability of human glioblastoma cells, U87, U373, and U251 expressing constitutively activated STAT3. We also investigated the inhibitory effects of LLL-3 on U87 glioblastoma cell growth in a mouse tumour model as well as the impact it had on the survival time of the treated mice. We observed that LLL-3 inhibited STAT3-dependent transcriptional and DNA binding activities. LLL-3 also inhibited viability of U87, U373, and U251 glioblastoma cells as well as induced apoptosis of these glioblastoma cell lines as evidenced by increased poly (ADP-ribose) polymerase (PARP) and caspase-3 cleavages. Furthermore, the U87 glioblastoma tumour-bearing mice treated with LLL-3 exhibited prolonged survival relative to vehicle-treated mice (28.5 *vs* 16 days) and had smaller intracranial tumours and no evidence of contralateral invasion. These results suggest that LLL-3 may be a potential therapeutic agent in the treatment of glioblastoma with constitutive STAT3 activation.

The signal transducer and activator of transcription (STAT) protein family is a group of primarily cytosolic proteins that play an important role in relaying signals from growth factors and cytokines (Zhong *et al*, 1994a, 1994b; Bromberg and Darnell, 2000; Shen *et al*, 2004). Several STATs have been associated with the induction and survival of cancer cells, however, STAT3 is especially prominent in this regard. The signal transducer and activator of transcription 3 is widely expressed in normal cells but its transient activation is strictly regulated by protein inhibitors of STATs (PIAS), SH2-containing tyrosine phosphatases (SHP1 and SHP2), and suppressor of cytokine signalling/extracellular signal-regulated kinase (SOCS/ERK). In several human cancers including glioblastoma, a dysregulation of the above mechanisms leads to constitutive activation of STAT3 (Rahaman *et al*, 2005). Constitutive STAT3 activation has been linked to cancer initiation, proliferation, promotion of angiogenesis and inhibition of apoptosis. There is evidence that constitutively active STAT3 can transform non-malignant cells into malignant cells (Huang *et al*, 2005). Activation of STAT3 has also been shown to enhance immune tolerance in glioblastoma, enabling cancer cells to evade immune surveillance (Hussain *et al*, 2007).

Given these oncogenic functions of STAT3, directly targeting the constitutive activation of the STAT3 pathway represents a potential therapeutic option in cancers with constitutively active STAT3. Activation of STAT3 occurs when it is phosphorylated at the Tyr-705 residue. This leads to dimerisation of STAT3 and subsequent transcription to the nucleus where further activation promotes DNA binding and translation of target genes (Carballo *et al*, 1999). Earlier findings have shown that STA-21, a polyphenol, can inhibit STAT3 activities and induce cell death in breast cancer cells (Song *et al*, 2005). Using a structure-based strategy, we selected a structural analogue of STA-21 termed as LLL-3 (Figure 1A), for further evaluation. Computer models with docking simulation showed that LLL-3 (Figure 1A) has a similar binding mode as STA-21, however, the smaller size of LLL-3 (molecular weight of 266 g mol^−1^ compared with the 306 g mol^−1^ of STA-21) would have an enhanced ability to transport into the cell.

Glioblastoma is the most common type of primary malignant brain cancer. Despite advances in surgical interventions, chemotherapy and radiation therapy, prognosis remains very poor, and current therapies are associated with significant side effects (Ries *et al*, 2004). Many clinical cases of glioblastoma and glioblastoma cell lines express constitutively activated STAT3 (Rahaman *et al*, 2002; Iwamaru *et al*, 2006). Studies have shown that inhibition of STAT3 reverses immune tolerance in malignant glioma cells (Hussain *et al*, 2007). These circumstances necessitate the search for novel targeted and more effective therapies for these cancers and STAT3 signalling represents an attractive target.

Here we report the inhibitory activity of LLL-3 on glioblastoma cell viability. We show that cell death occurs via apoptosis and also show that treating mice with intracranial human glioblastoma leads to a decrease in tumour size, invasiveness and prolongs their overall survival.

## Materials and methods

### Computational binding studies of LLL-3

In recent years, computational docking methods have been increasingly used to predict the binding of a ligand to a specific site of a protein structure. AutoDock4 is a docking program with a graphical tools package that uses a scoring function, also known as a force field, to search through a conformational landscape of a ligand to calculate the free energy of binding to a target site of a macromolecule. AutoDock4 determines the conformations of a ligand in the binding site while minimising the total energy of the protein–ligand complex, computed according to the following equations (Huey *et al*, 2007): 
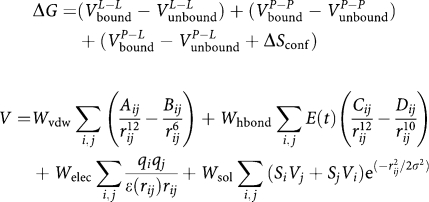
 ΔG refers to the change in free energy of Van der Waals, hydrogen bonding, electrostatic, desolvation, and torsional forces. The small molecule LLL-3 was docked using the Lamarckian genetic algorithm and most default parameters. The docking procedure involved the preparation of the ligand and macromolecule, the assignment of Gasteiger charges, and the identification of the torsional root and the two rotatable bonds of the ligand. An AutoGrid map was then pre-computed for all atom types in the ligand set. After 10 million energy evaluations were completed, all the resulting conformations of the ligand in the binding pocket of the macromolecule were clustered into groups according to their conformations and the docking results based on the predicted correct docking pose of the ligand. The AutoDock4 docking results with LLL-3 produced two major clusters with different binding conformations of the ligand. The correct binding mode is the second largest cluster, which has a −6.8 kcal mol^−1^ free energy of binding and comprises 10% of all the possible docking conformations.

### Cell lines

U251 and U373 human glioblastoma cell lines were kindly provided by Dr Sean Lawler (Comprehensive Cancer Center, the Ohio State University). U87 human glioblastoma cells were purchased from the American Type Culture Collection (Manassas, VA, USA). All cancer cells were maintained in Dulbecco's modified Eagle's medium (DMEM) supplemented with 10% foetal bovine serum, penicillin (100 U ml^−1^) and streptomycin (100 U ml^−1^). Normal human bladder smooth muscle cells were purchased from the Cambrex Corporation (East Rutherford, NJ, USA) and were grown in the media recommended by the company. The cell culture incubator was set at 37°C and aired with 5% CO_2_.

### Reconstitution of LLL-3

LLL-3 was synthesised in Dr Pui-Kai Li's laboratory (School of Pharmacy, The Ohio State University). The powder was dissolved in sterile DMSO to make a 20 mM stock solution. The stock solution was stored at −20°C and thawed just before usage.

### Establishment of STAT3-dependent luciferase reporter in MDA-MB-231 cancer cells

Plasmid pLucTKS3 contains seven copies of STAT3-binding site in TK minimal promoter (Turkson *et al*, 1998) and its activation specifically depends on STAT3 status in the cell environment. pLucTKS3 luciferase reporter plasmid was transfected into MDA-MB-231 cancer cell line using Lipofectamine 2000 reagent. The stable clones, which showed high luciferase activity, were selected for screening STAT3 inhibitors. The selected clones were exposed to LLL-3 at a final concentration of 20 *μ*M for 48 h, and luciferase activity was measured by using a Promega Luciferease kit (Madison, WI, USA) following manufacturer's instructions.

### STAT3 DNA binding activity

U373 glioblastoma and MDA-MB-231 cancer cells were treated with 20–50 *μ*M LLL-3 for 48 h. In all, 2 pmol of biotinylated STAT3 wild-type consensus DNA binding sequences was added to 5–10 *μ*g of U373 or MDA-MB-231 nuclear extract. TransFactor Universal STAT3 Specific Kit (Clontech Laboratories Inc., Mountain View, CA, USA; catalogue no. 631958) that permits flexible assay design for the rapid identification of STAT3 transcription factor DNA binding in cell extracts using an ELISA (enzyme-linked immunosorbent assay) format was used to determine the STAT3 DNA binding activity.

### Cell viability assay

A total of 5000 cells of U87, U251, and U373 were seeded per well in triplicates in 96-well plates. Plates were then incubated overnight. The next day serial dilutions of LLL-3 were prepared to achieve desired concentrations. Untreated and DMSO-treated cells were seeded in triplicates and served as controls. Cells were incubated at 37°C for 72 h. 3-(4,5-Dimethylthiazolyl)-2,5-diphenyltetrazolium bromide (MTT) viability assays were carried out after 72 h according to the manufacturer's protocol (Roche Diagnostics, Mannheim, Germany). 3-(4,5-Dimethylthiazolyl)-2,5-diphenyltetrazolium bromide assay measures cell viability based on mitochondrial conversion of MTT from a soluble tetrazolium salt into an insoluble formazan precipitate. After dissolving in *N*,*N*-dimethylformamide, it can be quantified by spectrophotometry. All experiments were repeated three times.

### Immunofluorescence staining for apoptosis

Immunostaining was performed as described earlier (Chen *et al*, 2007). Cleaved-caspase-3 (Asp175) antibody was purchased from Cell Signaling Technologies Inc. (Danvers, MA, USA). Secondary goat anti-rabbit IgG(H+L) Alexa Fluo^R^ 594 antibody was purchased from Invitrogen (Carlsbad, CA, USA). U87, U373, and normal human bladder smooth muscle cells were plated in six-well plates containing sterile cover slips and allowed to grow overnight. They were then treated with 20 *μ*M LLL-3 or DMSO. Controls with no treatment were also performed. After 48 h, the cells were fixed using methanol/acetone (1 : 1 volume) for 30 min followed by three washes with 1 × PBS. After the third wash, cover slips were transferred to a new six-well plate. Immunofluorescence staining for cleaved caspase-3 (Asp175) was performed. Nuclei were stained with DAPI. The fluorescence and phase contrast photographs were documented using LEICA OM-IRB inverted fluorescent microscope (Leica Microsystems Inc., Bannockburn, IL, USA) with an attached diagnostic RI-SE6 monochrome digital camera (Diagnostic Instrument Inc., Sterling Heights, MI, USA).

### Western blot analysis

U87, U251, and U373 glioblastoma cells were plated in 10 cm sterile dishes and left to grow overnight. They were then treated with 20 *μ*M LLL-3 or DMSO. Control plates with no treatment were also performed. After 48 h, cells were harvested at 4°C in cold harvest buffer and spun down at 4°C at 3000 r.p.m. for 5 min. Cell pellets were lysed in cold RIPA lysis buffer containing protease inhibitors. Protein concentrations were determined using the BCA protein assay as per the manufacturer's protocol (PIERCE Biotechnologies, Rockford, IL, USA). Cleaved-poly (ADP-ribose) polymerase (PARP) (Asp214) antibodies were purchased from Cell Signaling Technologies Inc. (Danvers, MA, USA). Western blot analysis was performed as per established procedure. Membranes were also probed with an antibody to GAPDH to ascertain protein loading of cellular proteins. Membranes were analysed with enhanced chemiluminescence plus reagents and scanned with the Storm scanner (Amersham Pharmacia Biotech Inc., Piscataway, NJ, USA).

### Animal subjects and intracranial studies

Female athymic nude mice (nu/nu) were purchased from the animal production area of The National Cancer Institute-Frederick Cancer Research and Development Center (Frederick, MD, USA). All animal manipulations were carried out in accordance with institutional guidelines under approved protocols. Actively growing U87 glioblastoma cells were injected at a concentration of 1 × 10^5^ cells in 5 *μ*l. Mice were anaesthetised with ketamine/xylazine mixture (114 : 17 mg kg^−1^) and a 0.5-mm burr hole was made 1.5–2 mm right of the midline and 0.5–1 mm posterior to the coronal suture through a scalp incision. Stereotaxic injection used a 10 *μ*l syringe (Hamilton Co., Reno, NV, USA) with a 30-gauge needle, inserted through the burr hole to 3 mm, mounted on a Just For Mice™ stereotaxic apparatus (Harvard Apparatus) at a rate of 2 *μ*l min^−1^. Three days later, when microscopic tumours grew and animals were asymptomatic, the mice were randomly split into two groups of five animals. One group received a single intratumoural injection of 50 mg kg^−1^ of LLL-3 administered stereotaxically. Animals losing >20% of their body weight or having trouble ambulating, feeding, or grooming were killed.

### Magnetic resonance imaging

Magnetic resonance imaging (MRI) was performed on a 7T small animal system (Brucker Biospin, Ettlingen, Germany), equipped with an actively shielded gradient set, with a maximum gradient strength of 400 mT m^−1^. Signal excitation and reception was accomplished with a 25-mm Litz RF coil. Conventional T2-weighted RARE images (TE=60 ms, TR=3000 ms, slice thickness=1 mm, matrix=256 × 256) were acquired in coronal (FOV=2.5 × 2.5 cm^2^) and axial (FOV=2.2 × 2.2 cm^2^) orientations to assess differences in anatomy.

### Tumour volume calculations

Tumour volumes were calculated using ImageJ. Contours were drawn on the T2-weighted images, based on image pixel intensities. In brief, the tumours were manually outlined using T2 data with freehand selection tool or the polygon selection tool. Then the ‘measure’ command generated an area for the region on the slice. The area was converted to volume by multiplying it times a conversion factor (converted to the actual voxel size), and the known width of the slices (1 mm). This was carried out for each slice, and then the separate slice volumes were added together for a total volume.

### Statistical analysis

GraphPad Prism (version 5, GraphPad Software Inc., San Diego, CA, USA) was used for statistical analysis. Survival curves were generated by Kaplan–Meier method. The log-rank test was used to compare the distributions of survival times, and a *P*-value of <0.05 was considered significant. Microsoft excel was used to analyse the cell viability data. Results were presented as bar charts with error bars as needed.

## Results

### LLL-3 inhibits STAT3-dependent transcriptional and DNA binding activities

**Figure 1:**
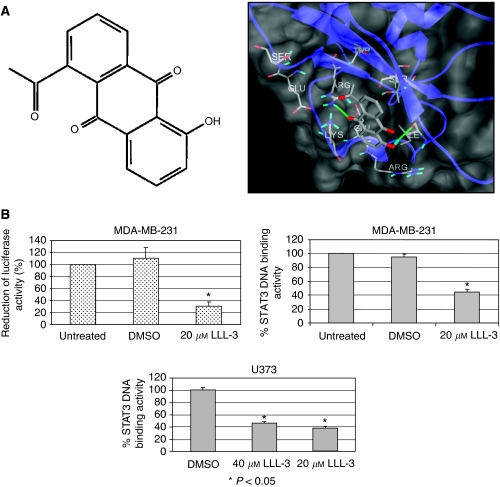
(**A**) AutoDock4 molecular surface visualisation of the STAT3 SH2 domain (residues 582–702) secondary structure ribbon, with selected binding pocket residues and predicted correct docking conformation of ligand LLL-3 (chemical structure insert), rendered by ball-and-stick models, in the target PTR-binding pocket of STAT3. The small molecule LLL-3, an analogue of STA-21, forms two hydrogen bonds with specific residues ARG609 and ILE634. (**B**) The inhibitory effect of STAT3 DNA binding activity in U373 and MDA-MB-231 cells and STAT3 transcriptional activity in MDA-MB-231 cells by LLL-3. Statistical significance (*P*<0.05) relative to the untreated (in MDA-MB-231 cells) or DMSO (in U373 cells) is designated by an asterisk.

We utilised a structure-based strategy to select LLL-3 (Figure 1A) as a STA-21 structural analogue and docking simulation showed a similar binding mode as STA-21. Because transcription and DNA binding activities are key functions for constitutively active STAT3 signalling in the promotion of oncogenesis, we examined whether LLL-3 could inhibit both STAT3's transcription and DNA binding activities. Our results show that LLL-3 inhibits STAT3-specific DNA binding activity in U373 glioblastoma cells and MDA-MB-231 breast cancer cells (Figure 1B) as well as inhibits STAT3-dependent transcription activity in luciferase assays in MDA-MB-231 breast cancer cells (Figure 1B).

### Inhibition of GBM cell viability by LLL-3

**Figure 2:**
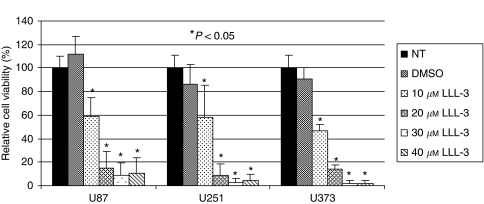
MTT cell viability assays in U87, U251, and U373 glioblastoma cells. Figure shows MTT cell viability after treatment with 20 and 30 *μ*M of LLL-3 and DMSO relative to untreated cells for 72 h. In U87, U251, and U373 glioblastoma cells, there is a decrease in viability after exposure to 20 *μ*M LLL-3. There is further decrease in viability with a treatment concentration of 30–40 *μ*M. Statistical significance (*P*<0.05) relative to the untreated (NT) is designated by an asterisk.

U87, U251, and U373 human GBM cells, which express constitutively active STAT3, were treated with 10–40 *μ*M of LLL-3. Our results showed that significantly decreased cell viability (<25%) was observed when compared with untreated cells and DMSO-treated cells. At a concentration of 30 *μ*M of LLL-3, cell viability decreased to <10% in U87, U251, and U373 glioblastoma cells (Figure 2). The decrease in cell viability was also visible through phase contrast microscopy as there are fewer cells when treated with LLL-3 (Figure 4).

### LLL-3 induced poly (ADP-ribose) polymerase cleavage in glioblastoma cells

**Figure 3:**
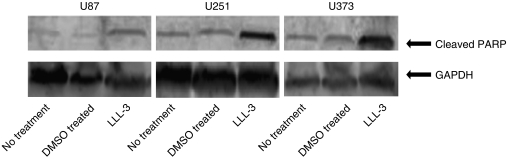
Western Blot analysis for cleaved PARP in U87 (left), U251 (middle) and U373 (right) glioblastoma cells. Cells were treated for 72 h. LLL-3-treated cells (right well in each gel) show PARP cleavage. There is minimal PARP cleavage in untreated and DMSO treated cells. For each cell line the signal for GAPDH (lower row) is about the same.

We next examined whether the inhibition of cell viability could be due to the induction of apoptosis in these glioblastoma cells. U87, U251, and U373 glioblastoma cells treated with 20 *μ*M of LLL-3 showed high levels of cleaved PARP (Asp214) whereas little PARP cleavage was observed in DMSO-treated and untreated cells as measured with western blot analysis (Figure 3). Analysis for GAPDH showed similar total protein amounts of GAPDH in the LLL-3, DMSO-treated and untreated samples. These results indicate that LLL-3 can induce apoptosis in these glioblastoma cells, which may account for the reduction of cell viability.

### LLL-3 induced caspase-3 cleavage

**Figure 4:**
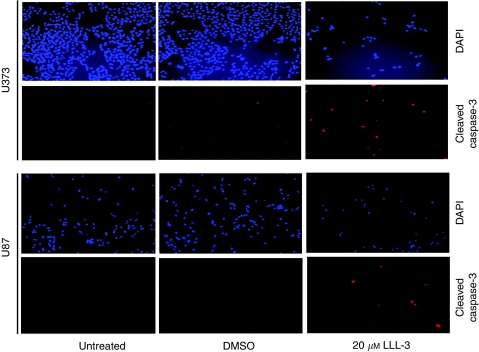
Immunofluorescence staining for cleaved caspase-3 in U373 and U87 glioblastoma cells after treatment for 48 h. Figure shows DAPI and red fluorescence at × 100 magnification. The clearly decreased cell density in the LLL-3-treated cells is evident. Most cells on the LLL-3-treated slide are cleaved caspase-3-positive (red fluorescence) as shown in Figure 4.

We also examined whether LLL-3 could induce caspase-3 cleavage, which contributes to the induction of apoptosis. U373 glioblastoma cells were treated with LLL-3, resulting in high levels of cleaved caspase-3 as measured by immunofluorescence staining. In contrast, DMSO-treated and untreated cells had very little cleaved caspase-3 (Figure 4). We also observed the induction of caspase-3 cleavage by LLL-3 in U87 human glioblastoma cells (Figure 4). However, the induction of cleaved caspase-3 was not observed in normal human bladder smooth muscle cells (data not shown), which do not express constitutively active STAT3.

### Intracranial tumour size and tumour infiltration

**Figure 5:**
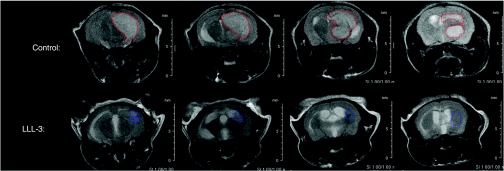
MRI image evaluation of U87 orthotopic glioblastoma model showed large tumours in the right frontal cortex of untreated control animals: midline shift was particularly evident (top row); LLL-3-treated animals showed tumours that were significantly smaller with decreased hyperintensity and no evidence of midline shift (bottom row). Volumetric analysis of the average tumour volume was estimated to be 17.2 mm^3^ for the untreated control mice and 17.0 mm^3^ for the LLL-3-treated mice.

Unilateral intracranial tumours were established in athymic nude mice and randomly treated with LLL-3 or control treated. Tumours in LLL-3-treated animals were significantly smaller and less hyper intense than tumours in control animals (Figures 5). We also detected distortion of the midline with tumour invasion into the contralateral side in control animals that was clearly not detected in the LLL-3-treated mice. Average tumour volume was calculated from sequential MRI images by analysis of individual pixel intensities to more accurately define tumour boundaries, and estimated to be 73.2 mm^3^ for control animals and 17.0 mm^3^ for LLL-3-treated animals, further showing that LLL-3 significantly contains tumour growth in an intracranial glioblastoma mouse model.

### Increased survival in LLL-3-treated animals

**Figure 6:**
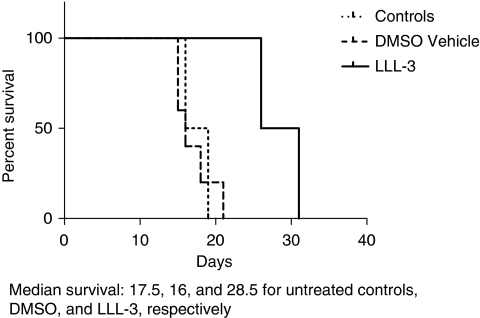
Kaplan–Meier survival analysis. Survival of mice with established brain tumours derived from U87 glioblastoma cells injected intracranially with 50 mg kg^−1^ of LLL-3 three days after implantation (*n*=5). Control mice with established brain tumours derived from U87 glioblastoma cells received no treatment (untreated control (*n*=5)). Survival was measured from the day of intracranial implantation of U87 glioblastoma cells. The median survival time of untreated control and DMSO vehicle-treated mice were 17.5 and 16 days, respectively whereas the LLL-3-treated mices’ survival was significantly increased to 28.5 days. The log-rank test was used to assess the statistical differences in survival among the two groups; *P*=0.03.

Animals receiving LLL-3 showed an initial small decrease in body weight (5–10%), which was quickly compensated for within three days following LLL-3 injection, and remained healthy for the duration of the study. The median survival time in the untreated and vehicle (DMSO)-treated animals were 17.5 and 16 days, respectively, compared with 28.5 days in the LLL-3-treated animals (*P*=0.03) (Figure 6). Prolonged survival time in LLL-3 treatment group was 190% compared with the control group. The increase in survival correlated with the tumour area as detected by MRI (Figure 6).

## Discussion

The signal transducer and activator of transcription 3 is an oncogenic protein that is capable of inducing tumours and enhancing cancer proliferation. In its inactive form, it is found predominantly in the cytoplasm. Phosphorylation at Tyr-705 causes it to dimerise and translocate to the nucleus where it binds to specific promoter sequences on its target genes. Blocking signalling to STAT3 by dominant-negative (DN) STAT3 mutant or antisense STAT3 oligonucleotides inhibits cancer cell growth, showing that STAT3 is crucial to the survival and growth of tumour cells (Kaptein *et al*, 1996; Aoki *et al*, 2003; Calvin *et al*, 2003). Constitutive STAT3 signalling participates in oncogenesis by stimulating cell proliferation, mediating immune evasion, promoting angiogenesis, and resistance to apoptosis induced by conventional therapies (Shen *et al*, 2001; Buettner *et al*, 2002; Real *et al*, 2002; Wang *et al*, 2004). There have been several strategies to inhibit the STAT3 pathway as a therapeutic approach in treating cancers, such as by using small molecules and DNA decoys (Lui *et al*, 2007). In addition, other inhibitors of STAT3 have also been reported including Cucurbitacin Q, which inhibits STAT3 phosphorylation (Sun *et al*, 2005), E804 that inhibits both Src and STAT3 (Nam *et al*, 2005); Stattic (Schust *et al*, 2006) and S3I-201 (Siddiquee *et al*, 2007), which inhibit STAT3 dimerisation, and SD-1029, which inhibits STAT3 nuclear translocation (Duan *et al*, 2006).

One difficulty with targeting proteins directly is that the targeted proteins may often also control other vital pathways sometimes leading to severe unwanted effects. LLL-3, a structural analogue of STA-21 with a smaller molecular weight and the added benefit of being easier to synthesise, may be a more suitable agent for targeting cancer cells with constitutively activated STAT3 pathway.

In this study, we evaluated the inhibitory efficacy of LLL-3 in human GBM cells. Our results show that, LLL-3 inhibits STAT3 activities, inhibits cell viability, and induces apoptosis in U87, U251, and U373 glioblastoma cells. We further show that LLL-3 inhibits tumour growth and inhibits tumour invasiveness as well as prolongs survival in an intracranial U87 glioblastoma xenograft mouse model. These results suggest that inhibition of STAT3 pathway may be an effective therapeutic approach for treating human glioblastoma.

Given that the STAT3 pathway may be necessary for renewal in some embryonic stem cells (Niwa *et al*, 1998), there is legitimate concern that indiscriminate inhibition of the STAT3 pathway may lead to side effects of some stem cells. However, as STAT3 phosphorylation in normal cells is transient, LLL-3 would likely have little effect on the proliferation of normal cells as suggested by its effect on normal bladder smooth muscle cells. Furthermore, studies using normal mouse fibroblasts showed that disrupting STAT3 signalling has much less profound effect in normal human and murine cells (Catlett-Falcone *et al*, 1999; Niu *et al*, 1999; Bowman *et al*, 2001; Burke *et al*, 2001; Song *et al*, 2005), suggesting that blocking STAT3 signalling may not be grossly toxic. Based on our findings, LLL-3 appears to be a potential therapeutic agent for human glioblastoma cells and possibly other tumours that have constitutively active STAT3. In addition to its apoptotic effects it may potentially render cancers more susceptible to other cytotoxic agents. It should be of interest to further explore the possibility of using LLL-3 as a potential agent for human glioblastoma treatment.

## References

[bib1] Aoki Y, Feldman G, Tosato G (2003) Inhibition of STAT3 signaling induces apoptosis and decreases survivin expression in primary effusion lymphoma. Blood 101: 1535–1542 12393476 10.1182/blood-2002-07-2130

[bib2] Bowman T, Broome MA, Sinibaldi D, Wharton W, Pledger WJ, Sedivy JM, Irby R, Yeatman T, Courtneidge SA, Jove R (2001) Stat3-mediated Myc expression is required for Src transformation and PDGF-induced mitogenesis. Proc Natl Acad Sci USA 98: 7319–7324 11404481 10.1073/pnas.131568898 PMC34666

[bib3] Bromberg J, Darnell Jr JE (2000) The role of STATs in transcriptional control and their impact on cellular function. Oncogene 19: 2468–2473 10851045 10.1038/sj.onc.1203476

[bib4] Buettner R, Mora L, Jove R (2002) Activated STAT signaling in human tumors provides novel molecular targets for therapeutic intervention. Clin Cancer Res 8: 945–954 11948098

[bib5] Burke W, Jin X, Liu R, Huang M, Reynolds RK, Lin J (2001) Inhibition of constitutively active Stat3 pathway in ovarian and breast cancer cells. Oncogene 20: 7925–7934 11753675 10.1038/sj.onc.1204990

[bib6] Calvin D, Nam S, Buettner R, Sekharam M, Torres-Roca J, Jove R (2003) Inhibition of STAT3 activity with STAT3 antisense oligonucleotide (STAT3-ASO) enhances radiation-induced apoptosis in DU145 prostate cancer cells. Int J Radiat Oncol Biol Phys 57: S297

[bib7] Carballo M, Conde M, El Bekay RE, Martin-Nieto J, Camacho MJ, Monteserin J, Conde J, Bedoya FJ, Sobrino F (1999) Oxidative stress triggers STAT3 tyrosine phosphorylation and nuclear translocation in human lymphocytes. J Biol Chem 274: 17580–17586 10364193 10.1074/jbc.274.25.17580

[bib8] Catlett-Falcone R, Landowski T, Oshiro M, Turkson J, Levitzki A, Savino R, Ciliberto G, Moscinski L, Fernandez-Luna J, Nunez G, Dalton W, Jove R (1999) Constitutive activation of STAT3 signaling confers resistance to apoptosis in human U266 myeloma cells. Immunity 10: 105–115 10023775 10.1016/s1074-7613(00)80011-4

[bib9] Chen C-L, Hsieh F-C, Lieblein JC, Brown J, Chan C, Wallace JA, Cheng G, Hall MB, Lin J (2007) Stat3 activation in human endometrial and cervical cancers. Br J Cancer 96: 591–599 17311011 10.1038/sj.bjc.6603597 PMC2360038

[bib10] Duan Z, Bradner JE, Greenberg E, Levine R, Foster R, Mahoney J, Seiden MV (2006) SD-1029 inhibits signal transducer and activator of transcription 3 nuclear translocation. Clin Cancer Res 12: 6844–6852 17121906 10.1158/1078-0432.CCR-06-1330

[bib11] Huang HF, Murphy TF, Shu P, Barton AB, Barton BE (2005) Stable expression of constitutively activated STAT3 in benign prostatic epithelial cells changes their phenotype to that resembling malignant cells. Mol Cancer 4: 2 15647107 10.1186/1476-4598-4-2 PMC546221

[bib12] Huey R, Morris G, Olson A, Goodsell D (2007) A semi-empirical free energy force field with charge-based desolvation. J Comput Chem 28: 1145–1152 17274016 10.1002/jcc.20634

[bib13] Hussain SF, Kong LY, Jordan J, Conrad C, Madden T, Fokt I, Priebe W, Heimberger AB (2007) A novel small molecule inhibitor of signal transducers and activators of transcription 3 reverses immune tolerance in malignant glioma patients. Cancer res 67: 9630–9636 17942891 10.1158/0008-5472.CAN-07-1243

[bib14] Iwamaru A, Szymanski S, Iwado E, Aoki H, Yokoyama T, Fokt I, Hess K, Conrad C, Madden T, Sawaya R, Kondo S, Priebe W, Kondo Y (2007) A novel inhibitor of the STAT3 pathway induces apoptosis in malignant glioma cells both *in vitro* and *in vivo*. Oncogene 26(17): 2435–2444 17043651 10.1038/sj.onc.1210031

[bib15] Kaptein A, Paillard V, Saunders M (1996) Dominant negative stat3 mutant inhibits interleukin-6-induced Jak-STAT signal transduction. J Biol Chem 271: 5961–5964 8626374 10.1074/jbc.271.11.5961

[bib16] Lui VW, Boehm AL, Koppikar P, Leeman RJ, Johnson D, Ogagan M, Childs E, Freilino M, Grandis JR (2007) Antiproliferative mechanisms of a transcription factor decoy targeting signal transducer and activator of transcription (STAT) 3: the role of STAT1. Mol Pharmcol 71: 1435–1443 10.1124/mol.106.032284 17325127

[bib17] Nam SBR, Turkson J, Kim D, Cheng JQ, Muehlbeyer S, Hippe F, Vatter S, Merz KH, Eisenbrand G, Jove R (2005) Indirubin derivatives inhibit Stat3 signaling and induce apoptosis in human cancer cells. Proc Natl Acad Sci USA 102: 5998–6003 15837920 10.1073/pnas.0409467102 PMC1087919

[bib18] Niu G, Heller R, Catlett-Falcone R, Coppola D, Jaroszeski M, Dalton W, Jove R, Yu H (1999) Gene therapy with dominant-negative Stat3 suppresses growth of the murine melanoma B16 tumor *in vivo*. Cancer Res 59: 5059–5063 10537273

[bib19] Niwa H, Burdon T, Chambers I, Smith A (1998) Self-renewal of pluripotent embryonic stem cells is mediated via activation of STAT3. Genes Dev 12: 2048–2060 9649508 10.1101/gad.12.13.2048 PMC316954

[bib20] Rahaman S, Harbor P, Chernova O, Barnett G, Vogelbaum M, Haque S (2002) Inhibition of constitutively active Stat3 suppresses proliferation and induces apoptosis in glioblastoma multiforme cells. Oncogene 21: 8404–8413 12466961 10.1038/sj.onc.1206047

[bib21] Rahaman SO, Vogelbaum MA, Hague SJ (2005) Aberrant stat3 signaling by interleukin-4 in malignant glioma cells: involvement of IL-13 Rα2. Cancer Res 65: 2956–2963 15805299 10.1158/0008-5472.CAN-04-3592

[bib22] Real P, Sierra A, De Juan A, Segovia J, Lopez-Vega J, Fernandez-Luna J (2002) Resistance to chemotherapy via Stat3-dependent overexpression of Bcl-2 in metastatic breast cancer cells. Oncogene 21: 7611–7618 12400004 10.1038/sj.onc.1206004

[bib23] Ries LAG, Melbert D, Krapcho M, Mariotto A, Miller BA, Feuer EJ, Clegg L, Horner MJ, Howlander N, Eisner MP, Reichman M, Edwards BK (2004) SEER cancer statistic Review 1975–2004. National Cancer Institute Bethesda, MD Huey R, Morris G, Olson A, Goodsell D (2007): A Semi-empirical Free Energy Force Field with Charge-Based Desolvation. J Comput Chem 28: 1145–1152

[bib24] Schust J, Sperl B, Hollis A, Mayer TU, Berg T (2006) Stattic: a small-molecule inhibitor of STAT3 activation and dimerization. Chem Biol 13: 1235–1242 17114005 10.1016/j.chembiol.2006.09.018

[bib25] Shen Y, Devgan G, Darnell JJ, Bromberg J (2001) Constitutively activated Stat3 protects fibroblasts from serum withdrawal and UV-induced apoptosis and antagonizes the proapoptotic effects of activated Stat1. Proc Natl Acad Sci USA 98: 1543–1548 11171987 10.1073/pnas.041588198 PMC29293

[bib26] Shen Y, Schlessinger K, Zhu X, Meffre E, Quimby F, Levy DE, Darnell Jr JE (2004) Essential role of STAT3 in post natal survival and growth revealed by mice lacking STAT3 serine 727 phosphorylation. Mol Cell Biol 21: 407–419 10.1128/MCB.24.1.407-419.2004 PMC303338 14673173

[bib27] Siddiquee K, Zhang S, Guida W, Blaskovich M, Greedy B, Lawrence H, Yip M, Jove R, McLaughlin M, Lawrence N, Sebti S, Turkson J (2007) Selective chemical probe inhibitor of Stat3, identified through structure-based virtual screening, induces antitumor activity. Proc Natl Acad Sci USA 104: 7391–7396 17463090 10.1073/pnas.0609757104 PMC1863497

[bib28] Song H, Wang R, Wang S, Lin J (2005) A low-molecular-weight compound discovered through virtual screening inhibits Stat3 function in breast cancer cells. Proc Natl Acad Sci USA 102: 4700–4705 15781862 10.1073/pnas.0409894102 PMC555708

[bib29] Sun J, Blaskovich M, Jove R, Livingston S, Coppola D, Sebti S (2005) Cucurbitacin Q: a selective STAT3 activation inhibitor with potent antitumor activity. Oncogene 24: 3236–3245 15735720 10.1038/sj.onc.1208470

[bib30] Turkson J, Bowman T, Garcia R, Caldenhoven E, De Groot RP, Jove R (1998) Stat3 activation by src induces specific gene regulation and is required for cell transformation. Mol Cell Biol 18: 2545–2552 9566874 10.1128/mcb.18.5.2545 PMC110634

[bib31] Wang T, Niu G, Kortylewski M, Burdelya L, Shain K, Zhang S, Bhattacharya R, Gabrilovich D, Heller R, Coppola D, Dalton W, Jove R, Pardoll D, Yu H (2004) Regulation of the innate and adaptive immune responses by Stat-3 signaling in tumor cells. Nat Med 10: 48–54 14702634 10.1038/nm976

[bib32] Zhong Z, Wen L, Darnell Jr JE (1994a) Stat3 and Stat4: members of the family of signal transducers and activators of transcription. Proc Natl Acad Sci USA 91: 4806–4810 7545930 10.1073/pnas.91.11.4806 PMC43877

[bib33] Zhong Z, Wen L, Darnell Jr JE (1994b) Stat3: a STAT family member activated by tyrosine phosphorylation in response to epidermal growth factor and interleukin-6. Science 264: 95–98 8140422 10.1126/science.8140422

